# Effect of Co-Production of Renewable Biomaterials on the Performance of Asphalt Binder in Macro and Micro Perspectives

**DOI:** 10.3390/ma11020244

**Published:** 2018-02-06

**Authors:** Xin Qu, Quan Liu, Chao Wang, Dawei Wang, Markus Oeser

**Affiliations:** 1 School of Transportation Science and Engineering, Harbin Institute of Technology, Harbin 150090, China; qu@isac.rwth-aachen.de; 2 Institute of Highway Engineering, RWTH Aachen University, D52074 Aachen, Germany; q.liu@isac.rwth-aachen.de (Q.L.); oeser@isac.rwth-aachen.de (M.O.); 3 Department of Road and Railway Engineering, Beijing University of Technology, Beijing 100124, China

**Keywords:** bio-asphalt binder, biomaterials, functional group, rheology, molecular dynamics simulation

## Abstract

Conventional asphalt binder derived from the petroleum refining process is widely used in pavement engineering. However, asphalt binder is a non-renewable material. Therefore, the use of a co-production of renewable bio-oil as a modifier for petroleum asphalt has recently been getting more attention in the pavement field due to its renewability and its optimization for conventional petroleum-based asphalt binder. Significant research efforts have been done that mainly focus on the mechanical properties of bio-asphalt binder. However, there is still a lack of studies describing the effects of the co-production on performance of asphalt binders from a micro-scale perspective to better understand the fundamental modification mechanism. In this study, a reasonable molecular structure for the co-production of renewable bio-oils is created based on previous research findings and the observed functional groups from Fourier-transform infrared spectroscopy tests, which are fundamental and critical for establishing the molecular model of bio-asphalt binder with various biomaterials contents. Molecular simulation shows that the increase of biomaterial content causes the decrease of cohesion energy density, which can be related to the observed decrease of dynamic modulus. Additionally, a parameter of Flexibility Index is employed to characterize the ability of asphalt binder to resist deformation under oscillatory loading accurately.

## 1. Introduction

Conventional hot mix asphalt (HMA) is produced with asphalt binder that derives from the petroleum refining process. However, the total storage of petroleum is limited and petroleum is non-renewable. Thus, it is necessary to develop an alternative binder material to entirely or partly replace the petroleum asphalt with respect to sustainable asphalt pavements. In recent years, biomass materials that can be derived from various bio-resources such as corn stover, animal waste wood and waste cooking oil are gaining interest from the pavement industry [1,2,3]. Significant research efforts are being made to develop “bio-asphalt binder or bio-binder” that incorporates the bio-oil as a new type of asphalt binder for future pavement construction [4,5,6,7,8,9,10,11,12,13,14,15,16,17]. Furthermore, Re-refined Engine Oil Bottoms (REOBs), which are one set of several products obtained in the refining of recovered engine oil, have been widely used in the asphalt industry since 1980 [18,19,20,21]. China is currently a large producer of waste cooking oil (WCO). Over 5 million tons of WCOs are generated every year. If the WCOs are not effectively recycled it may cause serious social and environmental problems. Currently the collected WCOs are mainly re-used for bio-diesel production. During this process about 10% heavy oil by-product is coproduced [22,23,24]. This black viscous liquid of WCO-based heavy oil residue has been regarded as the potential bio-oil modifier for producing WCO-based bio-asphalt. Now that the WCO is a promising biomass modifier for use as a possible asphalt substitute and replaces the conventional petroleum asphalt, several studies have been recently completed that are focused on the performance of bio-asphalt modified with WCO-based bio-oil. The effects of bio-oil obtained from WCO polymerization on conventional petroleum asphalt found that the addition of bio-oil increased thermal cracking resistance but reduced resistance to rutting according to Wen et al. [25]. Another result, stating that incorporating the WCO-based bio-oil into asphalt binder reduced the deformation resistance but improved the low temperature cracking resistance was reported by Sun et al. [26]. Recently, Azahar et al. proposed that the acid value of the WCO is a critical parameter for the performance of bio-asphalt. The permanent deformation resistance of bio-asphalt can be improved when the acid values of WCO are reduced by the chemical pre-treatments [27]. Since the addition of WCO-based bio-oil can soften the petroleum asphalt, many researchers also utilized this kind of bio-oil as a rejuvenating agent for the long-term aged asphalt binders from either the laboratory aging tests or actual field pavements [28,29,30,31,32,33].

Though the performance of WCO-based bio-asphalt has been comprehensively studied, there is still a lack of studies addressing the effects of WCO-based bio-oil on petroleum asphalt from a micro-scale perspective to understand the fundamental modification mechanism. Molecular Dynamics (MD) simulation is one of the effective analytical approaches to investigate the micro-structure and micro-properties of asphalt materials [34,35,36]. Bhasin et al. proposed the interrelationship between molecular properties, healing mechanisms and experimental parameters by means of MD simulations [37,38]. Xu et al. studied the cohesive and adhesive properties of asphalt concrete and evaluated the accuracy of modeling before and after aging through comparisons with experimental data [39,40]. Guo et al. investigated the asphalt micro-structure and micro-scale mechanical properties using the atomic force microscopy experiments and MD simulation and further studied the diffusion of different chemical components of asphalt on mineral aggregates surface [41,42]. Ding et al. studied the diffusion mechanism between the virgin binder and long-term aged binders from reclaimed asphalt pavements [43]. These examples indicate that the MD simulation is a powerful tool for investigating the modification mechanism of various asphalt modifiers.

The objective of this paper is to quantify the WCO-based bio-oil modification effect on the performance of petroleum asphalt from the micro-scale perspective. The molecular model of WCO is first created to represent bio-asphalt binders with various bio-oil contents. The validation of these molecular models is conducted by means of thermodynamic properties including the density, cohesive energy density and surface free energy. Then the bio-asphalt binders are simulated by means of a MD simulation to investigate several possible relationships between micro-scale characteristics and laboratory-tested macro-scale performance, which effectively reveals the micro-scale modification mechanism for the WCO-based bio-oil modified asphalt binders.

## 2. Materials and Performance Testing

### 2.1. Materials

#### 2.1.1. Asphalt Binder

In this study, a 60/80 penetration-grade asphalt binder was used as the control asphalt binder. This petroleum asphalt source was obtained from the Hebei province and has been widely applied to produce the HMA in the Beijing area. The physical properties of this control asphalt are shown in Table 1 in which all performance indices met the specification requirements in China.

#### 2.1.2. Bio-Oil

**Figure 1:**
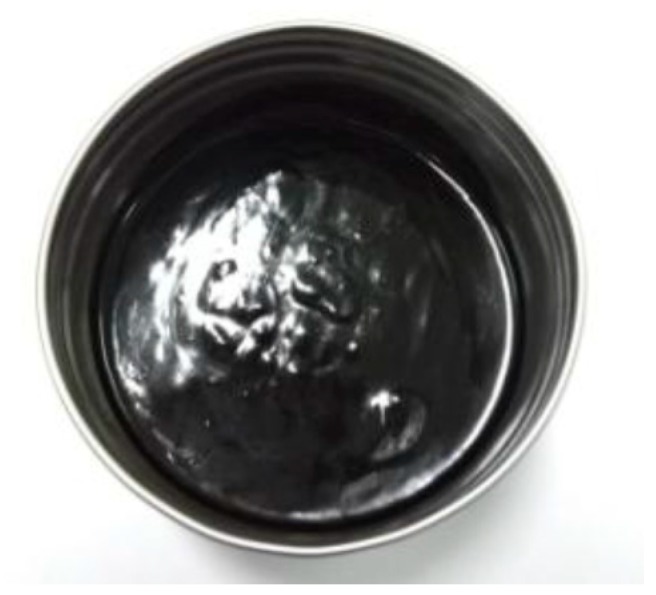
WCO-based bio-oil residue.

The bio-oil used in this study was a black oily liquid as shown in Figure 1, which was directly obtained from the WCO refining process for bio-diesel production. These bio-oil residue by-products were freely donated from the local commercial company that was engaged in collecting the WCOs and producing the bio-diesel. A distillation procedure under approximately 110 °C, which aimed at removing the presence of water and volatile contents, was performed for the bio-oil before adding it into the control asphalt as a modifier.

#### 2.1.3. Material Preparation

The control asphalt was first heated in the oven at a constant temperature of 150 °C for about one hour. Then 5%, 10%, and 15% bio-oil by weight of control asphalt binder was respectively added into the control asphalt when the temperature was stable at 140 °C. Afterwards a continuous 30 min of blending with a mixing speed of 4000 rounds per minute (rpm) using a high shear mixer followed to achieve a homogeneous mixing state. The sample IDs of the prepared asphalt binder materials are given in Table 2.

### 2.2. Performance Testing Procedures

#### 2.2.1. Fourier Transform Infrared Spectroscopy (FTIR) Test

The FTIR (BRUKER, Cologne, Germany) test has been an effective means for analyzing the chemical properties of asphalt binders. The chemical functional groups of asphalt can be identified based on the principle that the molecular rotation or oscillation at specific frequencies would result in the absorption of infrared spectra. Measuring the FTIR absorbance, the change in chemical functional groups of asphalt binder due to the incorporation of bio-oil residues can be detected and compared. The binder samples for FTIR tests were prepared by mixing asphalt binder with a common asphalt solvent of toluene followed by dropping the solution onto a KBr table. The toluene was then evaporated and the prepared sample was then scanned with the test spectrum range of 400 to 2000 cm^−1^.

#### 2.2.2. Complex Shear Modulus Test

The rheological tests in this study were completed with an Anton Paar MCR 302 dynamic shear rheometer (DSR, Anton Paar, Shanghai, China). The asphalt binder was a well-known viscoelastic material. The complex shear modulus of the oscillation loading was a measurement of the undamaged binder stiffness within the linear viscoelastic range using small strain amplitude, which was also dependent on the loading frequency and temperature. In this study, the complex shear modulus tests were performed under 1.58 rad/s at the temperature of 20 °C with the 8-mm parallel plate and 2-mm gap geometry.

#### 2.2.3. Linear Amplitude Sweep (LAS) Test

**Figure 2:**
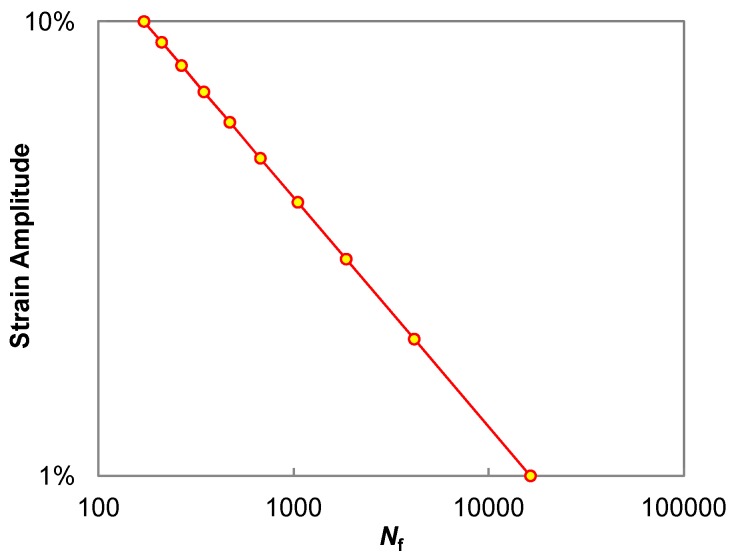
Fatigue life of asphalt binder from the LAS-based fatigue modeling.

Fatigue cracking due to repeated traffic loading is the main pavement distress that shortens the service life of asphalt pavements, which has been widely validated [48]. Based on the DSR equipment, the LAS test (AASHTO TP 101) was developed as the accelerated fatigue procedure for quantifying the damage resistance of asphalt binders [49,50,51] The LAS test uses an oscillatory strain sweep with the amplitudes linearly ranging from 0.1% to 30%. The data interpretation of the LAS test is based on the simplified-viscoelastic continuum damage (S-VECD) model from asphalt concrete fatigue modeling [52,53,54]. Details regarding to the recent developments of LAS-based fatigue modeling of asphalt binder are provided elsewhere [55,56]. As a result of modeling, the conventional fatigue life can finally be simulated and predicted under control-strain cyclic loading, the results of which are shown in Figure 2. The LAS testing temperature in this study was selected as 20 °C which represents the typical intermediate temperature in the Beijing area.

At least two replicates were run for all of the chemical and rheological performance tests to get the average test results. Three or even more replicates were completed to ensure the coefficient of variation was within 10 percent.

## 3. Performance Testing Results

### 3.1. FTIR Chemical Group Analysis

**Figure 3:**
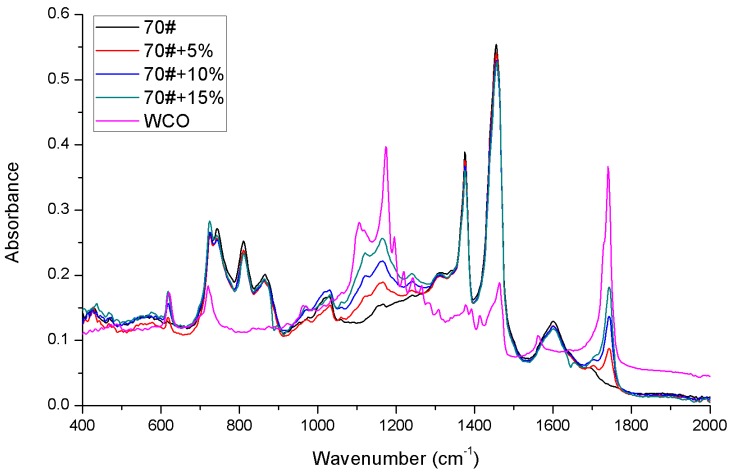
FTIR results for tested materials.

A step of baseline correction is conducted for the raw FTIR spectra of each binder to perform a reliable spectra comparison. The corrected FTIR spectra of control asphalt and bio-binders are shown in Figure 3. It can be observed that the three bio-binders generally display the same chemical functional groups to the control 70# binder. However, the distinguished absorbance of bio-oil for the functional groups of C-O stretch (1000–1300 cm^−1^) and C=O stretch (1735–1750 cm^−1^) exhibits much higher values than those of control 70# binder, indicating the existence of oxygen-related functional groups within the bio-oil. More specifically, it is also observed that the amount of C=O stretch and C–O stretch increases when increasing the content of bio-oil, which is expected from the spectra property of the bio-oil. Meanwhile, the S=O stretch (1030 cm^−1^) is also an important functional group for asphalt binder; however, the sulfoxide functional group within the control 70# binder and three bio-binders show slight differences.

To remove the variability of peak absorbance heights due to the various sample film thicknesses, the carbonyl index (C=O), carbon-oxygen index (C–O) and the sulfoxide index (S=O) developed by Lamontagne et al. were utilized to quantitatively describe the chemical structure change upon the bio-oil addition, as expressed in Equations (1)–(3) respectively [57].

(1) $$I_{C=O}=\frac{\mathrm{area}\;\mathrm{of}\;\mathrm{the}\;\mathrm{carbonyl}\;\mathrm{around}\;1700\;{\mathrm{cm}}^{-1}}{\mathrm{area}\;\mathrm{of}\;\mathrm{the}\;\mathrm{spectral}\;\mathrm{band}\;\mathrm{between}\;2000\;\mathrm{and}\;600\;{\mathrm{cm}}^{-1}}$$

(2) $$I_{C-O}=\frac{\mathrm{area}\;\mathrm{of}\;\mathrm{the}\;\mathrm{carbon}-\mathrm{oxygen}\;\mathrm{band}\;\mathrm{between}\;1300\;\mathrm{and}\;1000\;{\mathrm{cm}}^{-1}}{\mathrm{area}\;\mathrm{of}\;\mathrm{the}\;\mathrm{spectral}\;\mathrm{band}\;\mathrm{between}\;2000\;\mathrm{and}\;600\;{\mathrm{cm}}^{-1}}$$

(3) $$I_{S=O}=\frac{\mathrm{area}\;\mathrm{of}\;\mathrm{the}\;\mathrm{sulfoxide}\;\mathrm{band}\;\mathrm{around}\;1030\;{\mathrm{cm}}^{-1}}{\mathrm{area}\;\mathrm{of}\;\mathrm{the}\;\mathrm{spectral}\;\mathrm{band}\;\mathrm{between}\;2000\;\mathrm{and}\;600\;{\mathrm{cm}}^{-1}}$$

The quantified I_C=O_, I_C-O_ and I_S=O_ indices for the tested materials are given in Table 3. It is found that both the I_C=O_ and I_C-O_ indices of bio-oil are much higher than those of control 70# binder. When further adding the bio-oil to the 70# binder, the I_C=O_ and I_C-O_ are observed to be monotonically increased. However, the I_S=O_ index of all materials almost stays around a constant level, indicating that the S=O content within bio-oil is similar to that of the control asphalt.

### 3.2. Complex Shear Modulus

**Figure 4:**
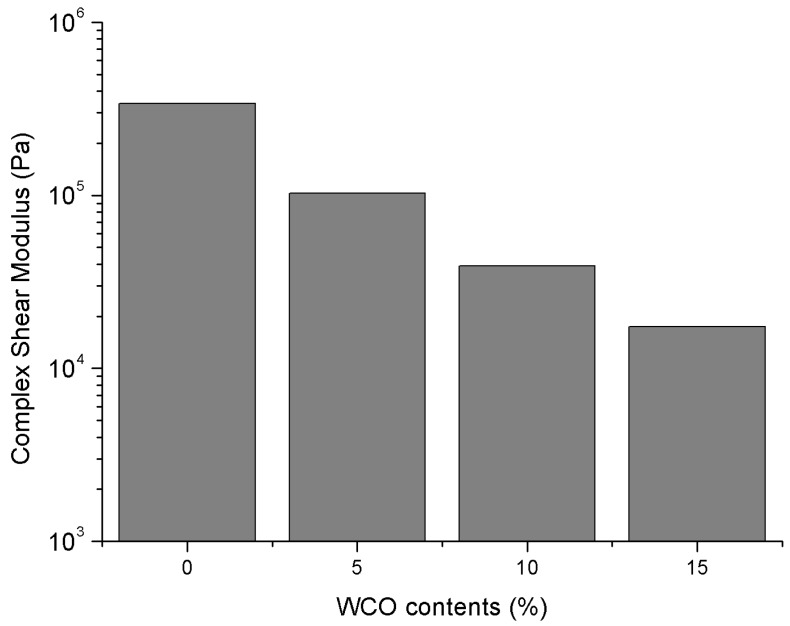
Complex shear modulus test results (20 °C, 1.58 rad/s).

The complex shear modulus at a temperature of 20 °C under a loading frequency of 1.58 rad/s for the control 70# binder and three bio-binders are presented in Figure 4. It is clearly seen that an increasing bio-oil content decreases the stiffness of the 70# binder, indicating that the bio-oil addition can soften the control asphalt binder. Since the WCO-based bio-oil residue in this study is shown as a viscous liquid at room temperature, the softening effects from the bio-oil addition are expected and also consistent to other previous WCO-based bio-asphalt studies described in the literature [25,26].

### 3.3. Fatigue Performance

**Figure 5:**
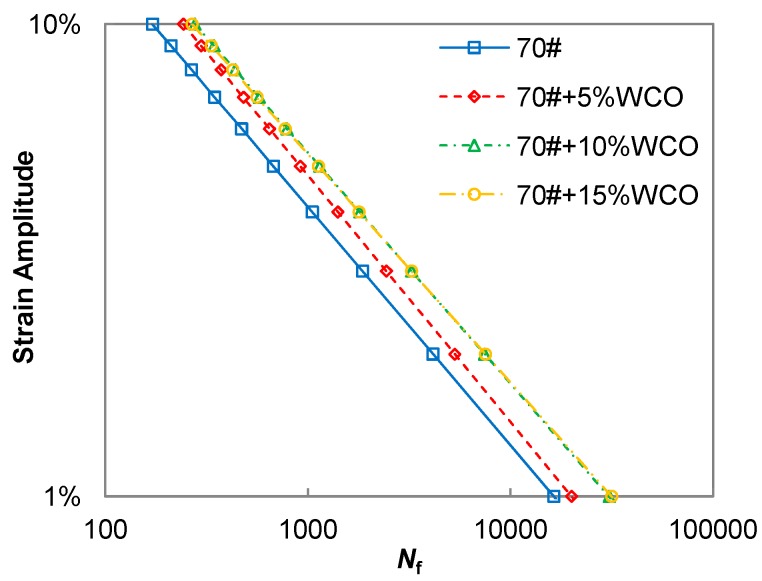
Fatigue life comparison between control 70# binder and three bio-binders.

The control 70# binder and three WCO-based bio-binders were subjected to the LAS test procedure followed by the fatigue performance analysis according to the S-VECD modeling approach [55,57]. The final modeling output is the classic fatigue law between the cyclic fatigue strain amplitude and corresponding simulated fatigue life as summarized in Figure 5. It can be observed that the binder fatigue life significantly increases with higher bio-oil weight indicating that the addition of bio-oil generally improves the fatigue resistance of the control 70# binder. However, two bio-binders of 70# + 10% WCO and 70# + 15% WCO exhibit identical fatigue live results under the strain amplitudes from 1% to 10%. The improvements of the fatigue performance of bio-asphalt binder still needs to be further validated by fatigue testing of bio-asphalt mixtures.

## 4. Creation and Validation of Molecular Structure Models

### 4.1. Molecular Structure Model of Asphalt Binder

**Figure 6:**
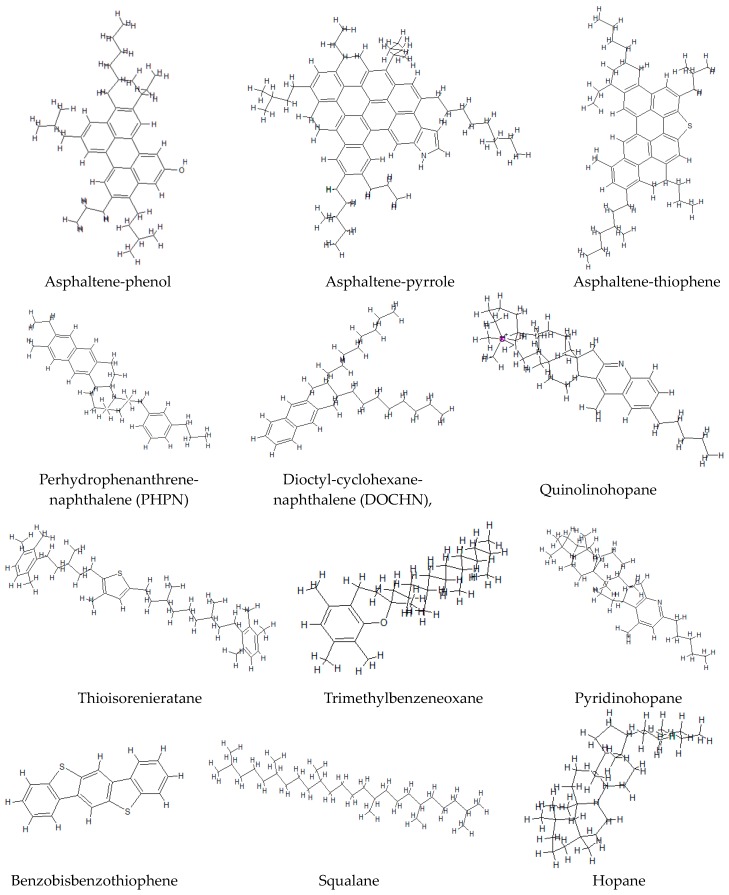
Typical microstructure for molecular structures of asphalt binder by SARA fractions [62,63,64,65,66,67,68,69,70,71,72].

Asphalt binder is well known as a typical organic material, which contains millions of complex chemical components [58]. In order to build the realistic and applicable asphalt binder molecular model, several researchers firstly separated asphalt binder into three, four or six fractions followed by the creation of a molecular structure for asphalt binder according to the respective proportions of each fraction [59,60]. In this paper, the 12-component molecular model of asphalt binder (see Figure 6), which was firstly proposed to represent SHRP core asphalt AAA-1, was utilized to represent the control 70# binder following the analysis approach that is detailed in a recent study of Li et al. [61]. Figure 1 shows the specific molecular structure of chemical components of asphalt binder following the SARA classification system (S for saturates, first A for asphaltene, R for resin and the second A for aromatics).

The unit numbers of the above 12 chemical components in the molecular system of asphalt binder are listed in Table 4 [61], based on this a typical molecular structure model for a given asphalt binder can be created.

### 4.2. Molecular Structure Model of WCO Based Bio-Oil

**Figure 7:**
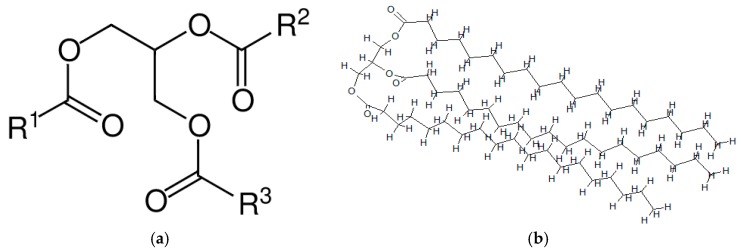
(**a**) Typical molecular unit of WCO-based bio-oil; (**b**) molecular structure of bio-oil.

Normally the WCO contains lots of complex chemical components due to its wide range of sources, so a feasible typical component should be selected to represent the WCO. The main chemical component within the WCO is the triglyceride (TG), which is an ester derived from glycerol and three fatty acids [73,74,75], and its content weight within WCO is more than 95% [76], therefore, the TG molecule is employed in this study to represent the micro-structure of the WCO-based bio-oil. The typical molecular structure unit is shown in Figure 7a, where R1, R2 and R3 represent the alkyl chains. The lengths of the three chains of fatty acids vary in naturally occurring TGs but normally contain 16, 18 or 20 atoms [76]. Therefore, the 18 carbon atoms are assigned into each chain to construct the TG molecule that represents the average molecular structure of WCO-based bio-oil as shown in Figure 7b.

**Figure 8:**
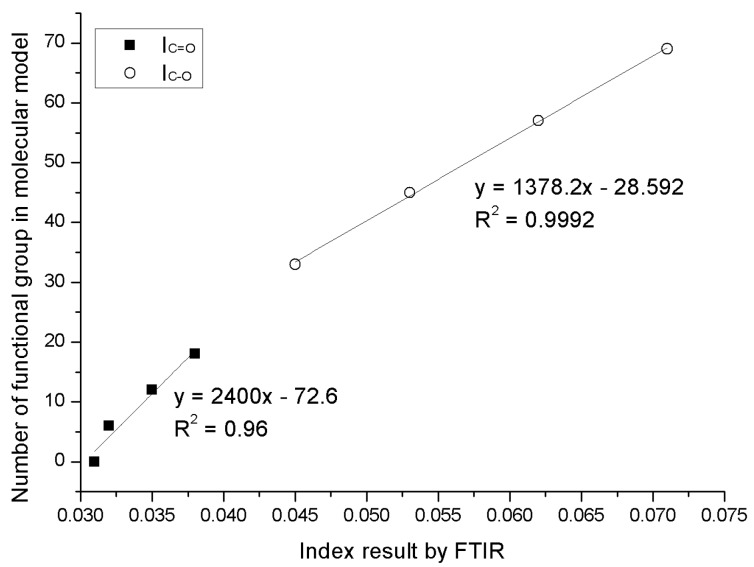
The correlation of C=O and C–O between molecular models and measured results.

The number of C=O and C–O within these created molecular structures for control 70# binder and three bio-binders are calculated to compare with the measured C=O and C–O by FTIR. The comparison results are shown in Figure 8. It can be seen that the number of the functional group in the asphalt binder model has a great correlation with previous obtained C=O and C–O functional group results of the bio-oil from the FTIR test, revealing the readability of the molecular asphalt binder models for the control 70# binder and three bio-binders.

### 4.3. Creation of Asphalt Binder Molecular Structure Model

To establish the molecular models of control 70# binder and three bio-binders respectively with 5%, 10% and 15% WCO-based bio-oil, four molecular models of asphalt binders with 0, 2, 4 and 6 WCO molecules are constructed according to the molar mass of the WCO (993.6 g/mol), and the number of WCO is also adopted as an integer. These four molecular structure models with different bio-oil contents are created by means of the software Material Studio 6.0 (Biovia, San Diego, CA, USA). The force field used in this study is the condensed-phase optimized molecular potential for atomistic simulation studies (COMPASS) force field, which is the first parameterized and validated first-principles-based force field and has been proved to be accurate for covalent molecules including most common organics, small inorganic molecules and polymers [77]. Then the molecular structure model is created by a geometry optimization since the molecules randomly appear in the system and the overlapping of molecules and extreme intermolecular interactions are inevitable. Many unreasonable molecular structures are eliminated during the geometry optimization process, and finally more appropriate relative positions of the molecules that follow the structural rules can be achieved. The MD simulation is then performed with a duration of 100 ps (1 ps = 10–12 s) under isothermal-isobaric (NPT) conditions to characterize the micro-scale properties of these established molecular structure models.

### 4.4. Validation of Molecular Structure Models

#### 4.4.1. Density

**Figure 9:**
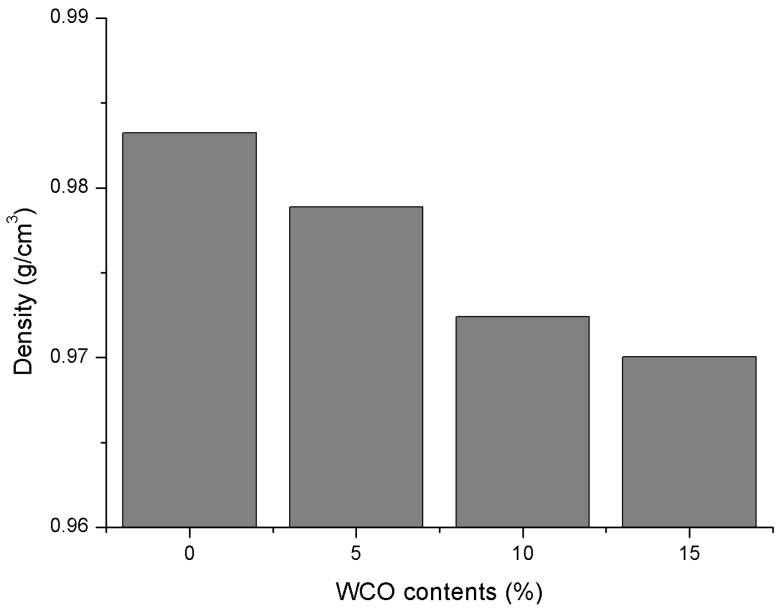
Density results of all binders with different WCO-based bio-oil contents.

The density information is first investigated to estimate the properties of these created molecular models from the MD simulation with duration of 100 ps under NPT conditions. The computation process took about 12 h and the simulated density results are given in Figure 9. It is seen that the binder density obviously decreases with the addition of WCO-based bio-oil. This is expected since the density of bio-oil is reported around 0.862 g/cm^3^ [78], which is lighter than the density of conventional petroleum-based asphalt. Therefore, the more the bio-oil is incorporated in the control asphalt, the lower binder density is obtained.

#### 4.4.2. Surface Free Energy

The parameter Surface Free Energy (SFE) measures the energy required to disrupt intermolecular chemical bonds when creating a new surface area [39]. In order to simulate the SFE properties, the bulk and confined models were respectively created for the control 70# binder and three bio-binders with various bio-oil contents. The SFE is defined and quantified as Equation (4) shows.

(4) $$\gamma_{a}=\left(E_{\mathrm{surface}}-E_{\mathrm{bulk}}\right)/2A$$

where γ_a_ is the SFE; E_surface_ and E_bulk_ represent the potential energy of the confined asphalt layer and bulk asphalt, respectively; and A is area of the new surface to be created.

**Figure 10:**
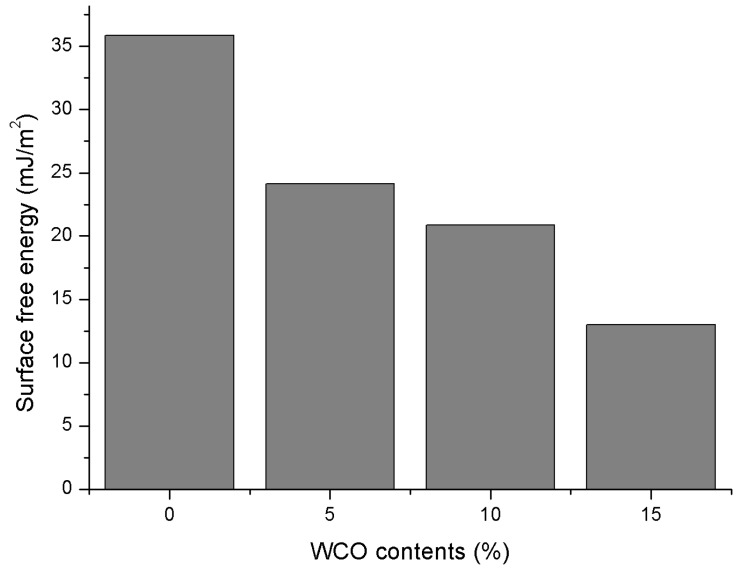
Surface free energy (SFE) of all binders with different WCO-based bio-oil contents.

As shown in Figure 10, the calculated SFE values of control 70# binder are larger than those of three bio-oil modified binders indicating the bio-oil addition causes a small surface free energy. Also, the SFE values decrease with an increase of bio-oil content. These SFE results for the created molecular structure models agree with the measured SFE values, ranging from 13 to 47.6 mJ/m^2^, as reported in the literature [79].

#### 4.4.3. Cohesion Energy Density

The Cohesive Energy Density (CED) is a mutual attraction between the molecules within the same material, which represents the manifestation of the molecular force between molecules of the same species and can be utilized to assess the intermolecular interaction inside an asphalt molecule model [80]. A series of material characteristics such as tensile strength, compressibility, coefficient of thermal expansion and wettability are all related to the material CED properties.

**Figure 11:**
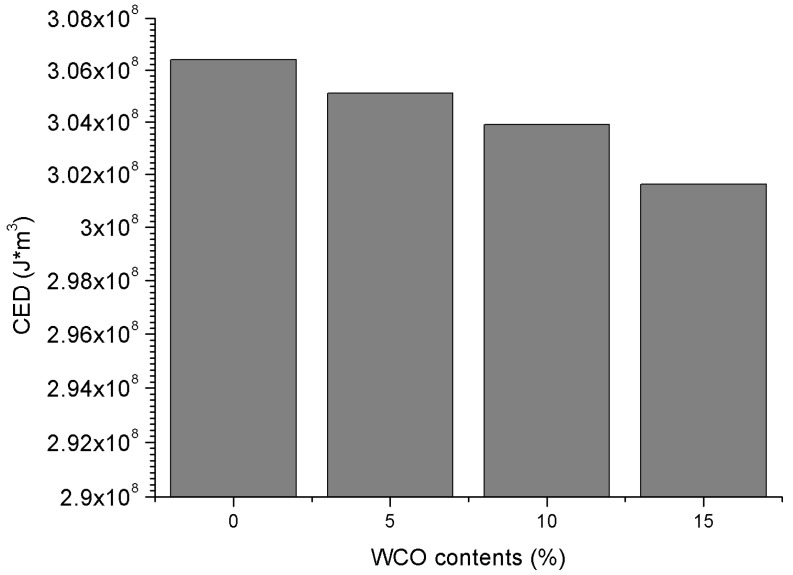
CED results of all binders with different WCO-based bio-oil content.

The CED results of the control 70# binder and three bio-binders are compared in Figure 11 and it is observed that the CED values decrease when increasing the bio-oil content. This is mainly due to the fact that the created bio-oil molecule has three long flexible molecular branches, which result in weaker intermolecular interactions compared to the control 70# binder.

The above MD simulation of density, SFE and CED properties validates that these created molecular structure models for control 70# binder and three bio-binders with different bio-oil contents are reasonable since the simulation results of these models are identical to the previous experimental results and research findings [40,79].

## 5. Relationship between Micro-Properties and Macro-Performance

### 5.1. CED vs. Dynamic Modulus

The CED parameter characterizes the cohesion energy within a unit volume. Previous research [80] showed that the CED could be determined by the viscosity of polymer, indicating that some scientific connection exists between the micro-scale CED and macro-scale viscosity for a given polymeric material. The asphalt binder is a typical viscoelastic material that shows a similar time-dependent behavior to the polymers. Therefore, it is also promising to link the CED property to the macro-scale performance of asphalt binders.

**Figure 12:**
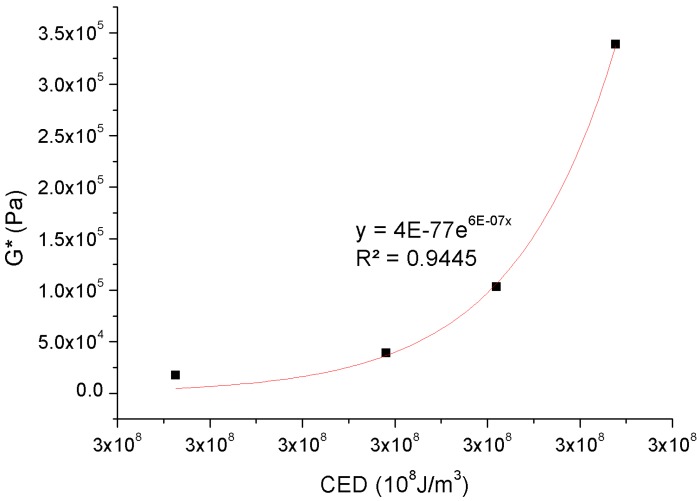
Relationship between micro-scale CED and macro-scale complex shear modulus.

The complex shear modulus represents the typical linear viscoelastic (LVE) material property of a non-damaged body. Figure 12 shows the correlation between the simulated micro-scale CED properties and the measured macro-scale complex shear modulus for the control 70# binder and three bio-binders, in which a clear exponential relationship can be fitted. This further supplement and validates the correlation between the micro-property and the macro-performance of polymer in the previous research [80]. In this case, the asphalt binder is subjected to oscillatory shear loading to measure the complex shear modulus response and thus, a larger micro-scale CED level means a stronger link between the molecules within asphalt binder, leading to a greater stability against shear deformation in the macro-scale.

### 5.2. Flexibility Index vs. Fatigue Life

Fatigue resistance of asphalt binder is important for fatigue performance of asphalt mixture and pavement. Currently main research efforts are focused on the fatigue performance evaluation and prediction based on laboratory testing. However, there are only limited studies addressing the fatigue damage and failure mechanisms from the micro-scale perspective. Essentially, the fatigue resistance of asphalt binder is mainly determined by various chemical properties such as the chemical composition, molecular weight, functional group and molecular structure. Therefore, investigating the micro-scale material property enables insightful understanding for comparing the distinguished fatigue resistance of different asphalt binders.

In Section 3.3, the fatigue performance of control 70# binder and three bio-binders are characterized using the LAS-based fatigue modeling approach to obtain the fatigue life results. When applying some deformation to the asphalt binder, the stiffest molecular chain would be the first to break, which is the source of fatigue cracks [81]. To investigate the micro-scale fundamental explanations for the improved binder fatigue life from the addition of bio-oil, a parameter of Flexibility Index (FI) is employed for a given molecular structure of asphalt binder. Kier [82] proposed this definition for FI based on multiple structural attributes from the molecular size, branching, cycles and heteroatom content. Each of the structural attributes in the definition has been incorporated into the analysis of shape using the kappa indices. In particular, the number of atoms and the relative periodicity of molecules are encoded into the K_1_ index; the branching or relative spatial density of molecules is encoded into the K_2_ index. The K_1_ and K_2_ indices are respectively derived as Equations (5) and (6).

(5) $$K_{1}=N{\left(N-1\right)}^{2}/P^{2}$$

(6) $$K_{2}=\left(N-1\right){\left(N-2\right)}^{2}/P^{2}$$

where N is the number of atoms; P is the number of paths with a length of one [82].

Then, the α-modified shape indices $K_{1}^{\alpha }$ and $K_{2}^{\alpha }$ are further calculated as Equations (7) and (8), which take into consideration the contribution made by covalent radii and hybridization to the shape of the molecule.

(7) $$K_{1}^{\alpha }=\left(N+\alpha \right){\left(N+\alpha -1\right)}^{2}/{\left(P+\alpha \right)}^{2}$$

(8) $$K_{2}^{\alpha }=\left(N+\alpha -1\right){\left(N+\alpha -2\right)}^{2}/{\left(P+\alpha \right)}^{2}$$

The modifier item of α is given by Equation (9).

(9) $$\alpha_{x}=r_{x}/r\left({\mathrm{Csp}}^{3}\right)-1$$

where $r_{x}$ is the radius of the *x*th heavy atom and $r\left({\mathrm{Csp}}^{3}\right)$ is the radius of sp^3^ carbon, which is assumed to be 0.77 Å [82].

Finally, the Flexibility Index (FI) can be calculated using Equation (10).

(10) $$ф=\frac{K_{1}^{\alpha }K_{2}^{\alpha }}{N}$$

where N is the number of non-hydrogen atoms in the molecule.

The FI parameters of the fraction structure are calculated and shown in Table 5.

**Figure 13:**
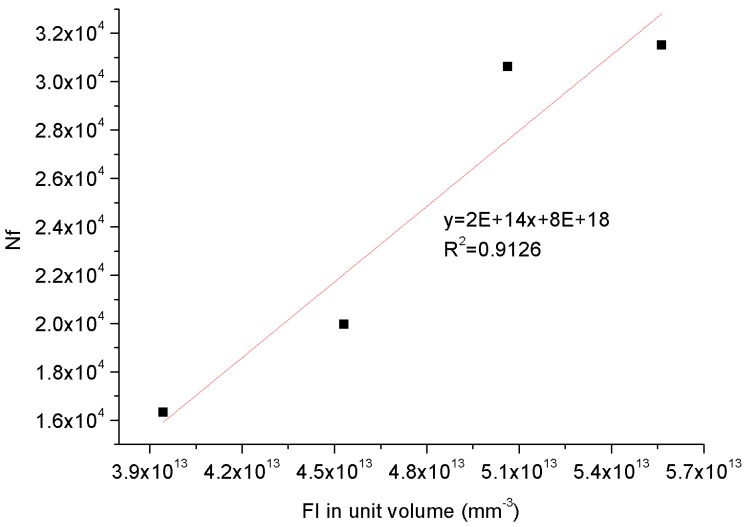
Relationship between micro-scale flexibility index in unit volume and macro-scale fatigue life.

Table 5 shows that the WCO-based bio-oil molecule displays the largest FI value, indicating that the bio-oil is the most flexible material among all of the compositions. Based on the specific FI value of each chemical composition, the FI for the control 70# binder and three bio-binders with various bio-oil contents are summed and the FI in unit volume is calculated according to the volume of asphalt binder models based on the MD simulation, then the FI in unit volume is plotted with the corresponding binder fatigue life under strain amplitude of 1% as shown in Figure 13. It is interesting to observe that the FI in unit volume is well correlated to the fatigue resistance for tested binders, revealing that the macro-scale fatigue performance of asphalt binder is fundamentally governed by the molecule flexibility from the micro-scale perspective. The more flexible the binder molecule behaves, the better fatigue resistance it performs during a fatigue test, which is also consistent to the previous research findings regarding fatigue damage of polymeric matrix composites [81].

## 6. Conclusions

This paper investigates the performance of WCO-based bio-asphalt binders from both rheological and molecular simulation approaches. The bio-oil effects on the chemical functional groups, complex shear modulus and fatigue performance of control asphalt is firstly characterized and then followed by the MD simulation to explain the modification mechanisms of bio-oil from a micro-scale perspective. The specific findings of this study are summarized as follows:

(1) From FTIR tests, it is demonstrated that the bio-oil modified binders displayed increased carbonyl index when increasing the bio-oil content, whereas the sulfoxide index almost exhibited the same level as that of the control asphalt. Further rheological performance tests indicated that the addition of bio-oil softened the stiffness and improved the fatigue resistance of the control asphalt.
(2) A reasonable averaged molecular structure for bio-oil is created based on previous research findings and further validated with the FTIR results and the micro-scale properties in terms of density, SFE and CED from the MD simulation.
(3) A larger CED at micro-scale level represents a stronger link between the molecules within asphalt binder to provide a better stability against shear deformation in the macro-scale. Therefore, the observed complex shear modulus decrease due to the bio-oil addition is well related to simulated CED properties for all tested binders. Meanwhile, a parameter of Flexibility Index (FI) is utilized to investigate the micro-scale fundamental mechanisms for the improved binder fatigue life from the addition of bio-oil. The more flexible the binder molecule is obtained, the better its fatigue resistance.

In further research, more material properties of WCO-based bio-oil modified asphalt binder still need to be covered from both macro-scale and micro-scale. Also, the long-term aging effects on these material properties need to be addressed.

## Figures and Tables

**Table 1:** Physical properties of control asphalt.

Properties	Standard Test Method	Test Results
Penetration at 25 °C/0.1 mm	ASTM D5 [44]	75
Softening point/°C	ASTM D36 [45]	49
Ductility at 5 °C/mm	ASTM D113 [46]	35.5
Viscosity at 135 °C/Pa s	ASTM D4402 [47]	0.35

**Table 2:** Summary of asphalt binders.

Materials	Binder ID	Percent Weight of Bio-Oil Addition
Control 60/80 asphalt binder	70#	-
WCO-based Bio-oil modified asphalt binders	70# + 5% WCO	5% WCO
	70# + 10% WCO	10% WCO
	70# + 15% WCO	15% WCO

**Table 3:** Calculation results of functional groups indices.

Binder ID	I_C=O_	I_C-O_	I_S=O_
70#	0.031	0.045	0.030
70# + 5% WCO	0.032	0.053	0.027
70# + 5% WCO	0.035	0.062	0.030
70# + 5% WCO	0.038	0.071	0.028
WCO	0.058	0.225	0.032

**Table 4:** Molecules number in the model system.

Molecule	Number	Molecule	Number
Squalane	4	DOCHN	13
Hopane	4	Quinolinohopane	4
Asphaltene-phenol	3	Thioisorenieratane	4
Asphaltene-pyrrole	2	Trimethylbenzeneoxane	5
Asphaltene-thiophene	3	Pyridinohopane	4
PHPN	11	Benzobisbenzothiophene	15

**Table 5:** Flexibility index of each molecular structure.

Molecule	Flexibility Index	Molecule	Flexibility Index
Squalane	19.67	Quinolinohopane	6.13
Hopane	6.87	Thioisorenieratane	13.22
Asphaltene-phenol	12.03	Trimethylbenzeneoxane	9.92
Asphaltene-pyrrole	8.76	Pyridinohopane	6.11
Asphaltene-thiophene	9.01	Benzobisbenzothiophene	2.07
PHPN	6.50	WCO	55.53
DOCHN	10.27	-	-
